# Enhancement of Ceramics Based Red-Clay by Bulk and Nano Metal Oxides for Photon Shielding Features

**DOI:** 10.3390/ma14247878

**Published:** 2021-12-19

**Authors:** Mohamed Elsafi, Mirvat Fawzi Dib, Hoda Ezzelddin Mustafa, M. I. Sayyed, Mayeen Uddin Khandaker, Abdullah Alsubaie, Abdulraheem S. A. Almalki, Mahmoud I. Abbas, Ahmed M. El-Khatib

**Affiliations:** 1 Physics Department, Faculty of Science, Alexandria University, Alexandria 21511, Egypt; mirvatdib2018@gmail.com (M.F.D.); mabbas@physicist.net (M.I.A.); elkhatib60@yahoo.com (A.M.E.-K.); 2 Khalifa Medical Center, Abu-Dhabi 60843, United Arab Emirates; hezzeddin68m@gmail.com; 3 Department of Physics, Faculty of Science, Isra University, Amman 11622, Jordan; dr.mabualssayed@gmail.com; 4 Department of Nuclear Medicine Research, Institute for Research and Medical Consultations (IRMC), Imam Abdulrahman bin Faisal University, P.O. Box 1982, Dammam 31441, Saudi Arabia; 5 Center for Applied Physics and Radiation Technologies, School of Engineering and Technology, Sunway University, Bandar Sunway 47500, Selangor, Malaysia; mayeenk@sunway.edu.my; 6 Department of Physics, College of Khurma, Taif University, P.O. Box 11099, Taif 21944, Saudi Arabia; a.alsubaie@tu.edu.sa; 7 Department of Chemistry, Faculty of Science, Taif University, Taif 21974, Saudi Arabia; almalki.a@tu.edu.sa

**Keywords:** red clay, bulk metal oxides, nano-CdO, MAC, *Z_eff_*

## Abstract

We prepared red clays by introducing different percentages of PbO, Bi_2_O_3_, and CdO. In order to understand how the introduction of these oxides into red clay influences its attenuation ability, the mass attenuation coefficient of the clays was experimentally measured in a lab using an HPGe detector. The theoretical shielding capability of the material present was obtained using XCOM to verify the accuracy of the experimental results. We found that the experimental and theoretical values agree to a very high degree of precision. The effective atomic number (*Z_eff_*) of pure red clay, and red clay with the three metal oxides was determined. The pure red clay had the lowest *Z_eff_* of the tested samples, which means that introducing any of these three oxides into the clay will greatly enhance its *Z_eff_*, and consequently its attenuation capability. Additionally, the *Z_eff_* for red clay with 10 wt% CdO is lower than the *Z_eff_* of red clay with 10 wt% Bi_2_O_3_ and PbO. We also prepared red clay using 10 wt% CdO nanoparticles and compared its attenuation ability with the red clay prepared with 10 wt% PbO, Bi_2_O_3_, and CdO microparticles. We found that the MAC of the red clay with 10 wt% nano-CdO was higher than the MAC of the clay with microparticle samples. Accordingly, nanoparticles could be a useful way to enhance the shielding ability of current radiation shielding materials.

## 1. Introduction

Most countries around the world consider nuclear technology to be an alternative energy source to solve the problem of nonrenewable energy, which will run out one day. Due to the increased use of radioisotopes and radiation-emitting devices in various medical and industrial fields, it is necessary to study the ability of some readily available materials for use in construction, such as concrete, rocks and clay, to protect against gamma rays [1,2,3,4].

It is well known that materials with a high atomic number and density are very useful as ionizing radiation shields. The most common materials used for these purposes are lead, alloys, glasses, composites, some types of concrete, and clay materials [5,6,7,8].

Clay has been used since antiquity, in Mesopotamia, Egypt, Africa, and the Middle East; and more recently in Roman and Islamic civilizations in Asia, North America, Medieval Europe, and so on. Civilizations have built entire cities out of clay materials. Clay products are now used by more than a third of the world’s population due to their high quality and resistance to weathering. Clay material in architecture is a part of the heritage of almost every nation on every continent.

In many developed and developing countries, clay materials are used for building and construction. Clay products, such as ceramic pots, fired bricks, and tiles (for ceilings and floors) are less expensive and more durable than cement, and they are environmentally friendly and safe building materials that are widely available at low prices in various regions [9,10,11]. Furthermore, clay has refractory properties such as a high melting point, thermochemical stability, abrasion resistance, and thermal shock resistance. One of the most important characteristics that distinguishes clay and from other materials is that it is non-toxic. Due to these characteristics, clay materials are suitable for use as shielding materials (designing radiation shields from clay materials) [12,13,14].

With the emergence of nanotechnology as a progressive branch of science in recent years, various types of nanoparticles have been used to design radiation shields. The advantage of using nanomaterials in this field is that the distances between molecules are very small, increasing the possibility of photon collisions with atoms of the material, and thus improving the material’s ability to attenuate photons [15,16,17,18].

Nuclear engineers have placed a high value on nanocomposites containing metals or oxides of heavy elements, and their research has focused on developing these nanocomposites for use as an alternative to traditional radiation shields due to their promising properties, such as their lightweight, and desirable mechanical, chemical, and physical properties [19,20]. The majority of previous research has focused on developing some types of clay mixed with some heavy oxides for use as radiation shields, but very few studies have focused on developing clays mixed with nano-scale particles of heavy oxides [21].

Some types of Egyptian clay, which are natural building materials that may be considered for use as a radiation-shielding materials, will be investigated in this study. Clay can be found in relatively large reserves northeast of the city of Aswan in Egypt. Many companies produce it for the local ceramics and tile industries, mainly in Wadi Abu Sabira and Wadi Abu Ajaj. Due to the industrial importance of Aswan clay, some technical studies have been conducted to investigate its physical properties, either in its raw state as used for the manufacture of ceramics and tiles, or as a mixture with other raw materials [22].

Red clay is a clean and environmentally friendly building material that can be used as a radiation shield in radiation protection applications, or it can be added to concrete mixtures in certain proportions as an alternative to sand, resulting in an increase in its density, which leads to an increase in gamma ray attenuation. This form of clay’s high melting point is indicative of its potential thermal stability in the case of prolonged exposure to high-energy radiation, and its compressive strength is appropriate for the production of high-strength shielding materials [23].

However, to the best of the authors’ knowledge, studies related to the radio protective properties of these clays are almost non-existent, which prompted the researchers in this work to study the radio protective properties of red clay found in the Aswan region of Egypt, after adding a group of heavy-metal oxides as both micro- and nano-scale particles. In this work, some red clay originating from ceramic samples was prepared and the chemical composition was deduced by EDX analysis. The attenuation parameters of these samples were experimentally determined and compared with theoretical values produced by the XCOM software. The effective atomic number (*Zeff*) was calculated for a broad energy range.

## 2. Materials and Methods

First, the red clay samples were collected from Aswan city, Egypt, then dried, crushed, and sieved using a sieve with a hole diameter of 100 μm. Secondly, micro-scale metal oxides (PbO, Bi_2_O_3_ and CdO) were purchased from the El-Gomhouria Company in Egypt. The average particle size of these oxides ranged from 50 to 100 µm, and their purity was up to 99%. Meanwhile, nano-scale cadmium oxide (CdO) particles (average size 40 nm) were purchased from the NanoTech Company in Egypt, where they were chemically prepared. The red clay was mixed with the proportions of oxides shown in Table 1, and blended well by a mixer to obtain a homogeneous mixture. This mixture of powders was added to a proportion of water (mixture: water = 3:1) to form the compound, then put in a plastic container and allowed to dry for two weeks. Thus, 10 samples were prepared.

**Figure 1:**
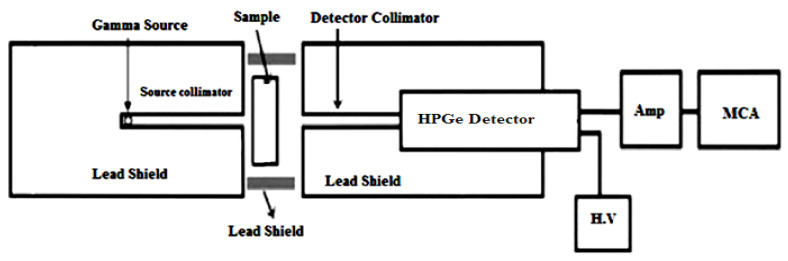
The schematic diagram of the experimental setup for the narrow-beam method.

These samples were left for two weeks to dry, and a sample of each type was then taken to measure its chemical composition by energy dispersive X-ray (EDX) analysis, as shown in Table 1. From knowledge of their compositions, the MAC could be theoretically calculated using the WinXCom program [24,25,26]. The radiation shielding parameters were experimentally determined by the narrow-beam method. A high-purity germanium (HPGe) detector was used, alongside point sources of different energies in cases where their activities and other specifications could be found (Table 2) [27,28,29,30,31,32,33]. The sample was placed between the source and the detector using a collimator and lead shield. The schematic diagram of the experimental measurement technique is shown in Figure 1. Measurements were undertaken for a time sufficient for the statistical uncertainty of the area under the peak to be less than 1%, and the count rate was calculated in the presence and absence of the sample. The MAC is calculated according to the following equation [34,35,36]:

(1) $$\mathrm{MAC}=\frac{1}{x\cdot \rho }\ln\frac{A}{A_{O}}$$

where *A* and *A*_0_ represent the areas under the peak, and the count rates obtained from the spectrum in the presence and absence of the absorbing sample, respectively, × (cm) represents the thickness of the measured clay sample, and *ρ* (g/cm^3^) the density. The linear attenuation coefficient or LAC is defined as the probability of photons interacting with matter per unit path length, and was calculated to determine other important shielding parameters (such as HVL and TVL) where the LAC equals MAC*ρ. The HVL and TVL represent the thicknesses needed to attenuate 50% and 90% of the initial photon intensity, respectively, and can be evaluated by the following equations [37,38]:

(2) $$\mathrm{HVL}=\frac{\ln2}{\mathrm{LAC}}\;,\;\mathrm{TVL}=\frac{\ln10}{\mathrm{LAC}}$$

The effective atomic number (*Z_eff_*) is another useful radiation interaction factor that is used to describe the attenuating properties of mixtures or compounds in terms of the elements present, and depends on the incoming photon energy. *Z_eff_* values for the studied polymers can be obtained using Equation (3) [39]:

(3) $$Z_{eff}=\frac{\sum_{i}f_{i}A_{i}{\left(\mathrm{MAC}\right)}_{i}}{\sum_{j}\frac{A_{j}}{Z_{j}}{\left(\mathrm{MAC}\right)}_{j}}$$

where *f_i_*, *Ai*, and *Z_i_* refer to the mole fraction, atomic weight, and an atomic number of each constituent element in the selected polymer, respectively.

## 3. Results and Discussion

**Figure 2:**
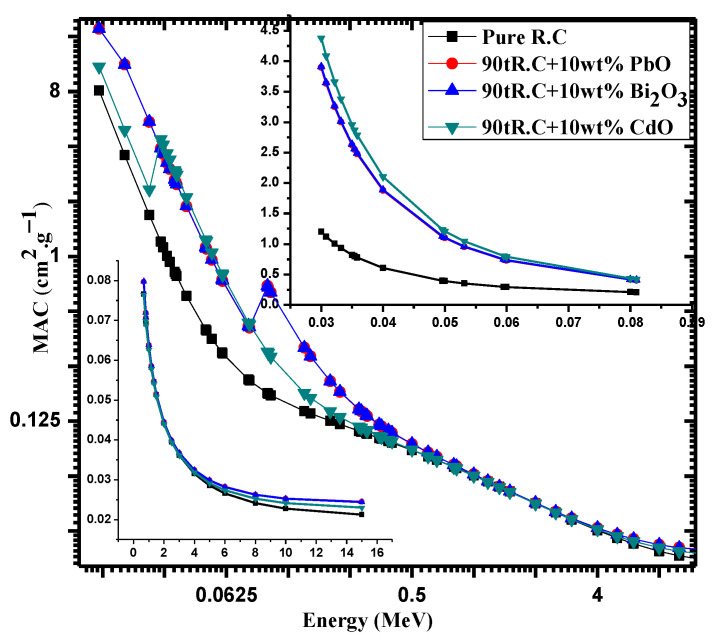
The MAC as a function of energy ranging from 0.015–15 MeV for different micro-samples calculated by XCOM software.

Figure 2 shows the mass attenuation coefficient (MAC) for the tested clays with different micro-samples as a function of energy between 0.015 and 15 MeV. The values were calculated using the XCOM software. In this work, red clay, which is used as a building material, was prepared using three oxides: PbO, CdO, and Bi_2_O_3_. In Figure 2, 10 wt% PbO, CdO, and Bi_2_O_3_ was added to the red clay, and the figure presents the effect of this addition on MAC. In the low-energy region (energies less than 70 MeV), it can be seen that the red clay with 10 wt% CdO has a greater MAC than the red clay with PbO and Bi_2_O_3_. This difference is due to the k-absorption edges of Cd, Pb, and Bi, which occur at 26.71, 88, and 90.53 keV, respectively. Due to Cd’s k-absorption edge, it has a high attenuation ability, near 20–30 keV, causing it to have a higher MAC value than PbO and Bi2O3. Meanwhile, as the energy approaches 80 keV, the k-absorption edges of Pb and Bi cause the clays with these two elements to have a higher MAC than the clay with CdO.

**Figure 3:**
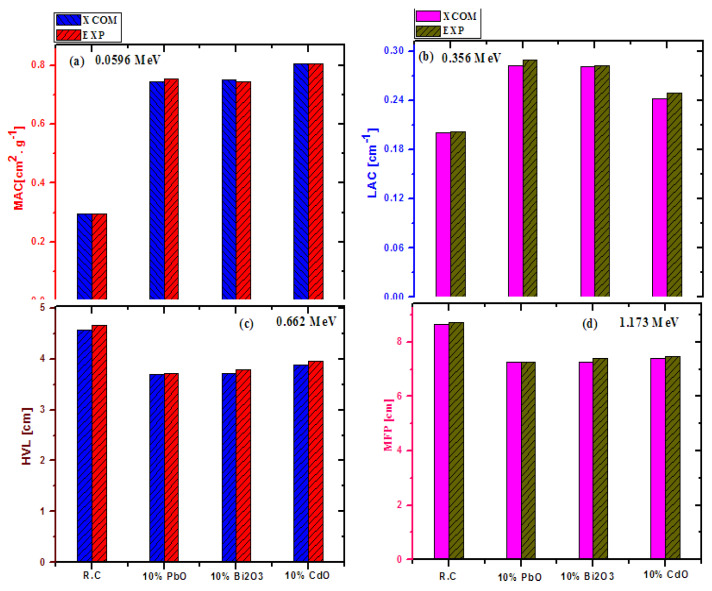
The difference between the experimental and XCOM results of (**a**) MAC at 0.0596 MeV, (**b**) LAC at 0.356 MeV, (**c**) HVL at 0.662 MeV and (**d**) MFP at 1.173 MeV for red clay, as well as different 10% wt doped oxide-clays.

In order to understand the influence of introducing PbO, Bi_2_O_3_, and CdO into red clay on its attenuation ability, the MAC and LAC of the clays were experimentally measured in a lab, and from these experimental values the HVL, TVL, and MFP ewere determined. Before analyzing the shielding ability of the clays, it is important to verify the accuracy of the experimental results, as all the conclusions rely on it. For this, the theoretical shielding capability of a material is obtained using XCOM, and then these results are compared with the experimental data. The theoretical results of red clay (no additives), red clay with 10 wt% PbO, red clay with 10 wt% Bi_2_O_3_, and red clay with 10 wt% CdO were compared at four different energies, as shown in Figure 3. All four tested parameters (MAC, lAC, HVL, and MFP) had a good level of agreement between their experimental and theoretical results, at all energies, and for all tested samples. For instance, the difference between the experimental and theoretical MAC for red clay with 10 wt% CdO at 0.0596 MeV is negligible, meaning that the two values agree to a very high degree of precision. The same results were found for the other samples and the other tested parameters. This result proves that the experimental setup used in this study can be reliably used to determine the shielding ability of the investigated clays.

**Figure 4:**
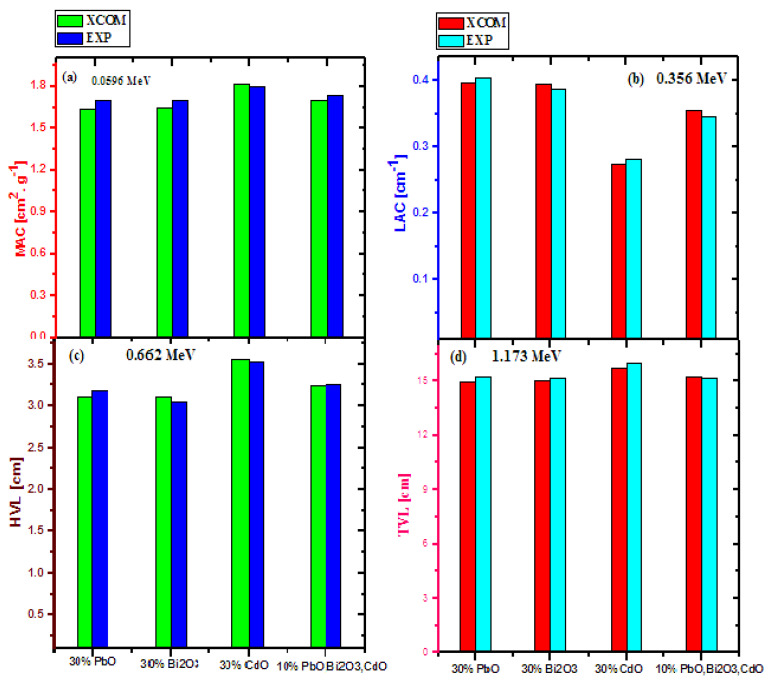
The difference between the experimental and XCOM results of (**a**) MAC at 0.0596 MeV, (**b**) LAC at 0.356 MeV, (**c**) HVL at 0.662 MeV and (**d**) TVL at 1.173 MeV for different 30% wt doped oxide-clays.

Figure 4 shows the difference between the experimental and simulated XCOM results of four different parameters at four selected energies. To ensure the validity of the experimental results, the accuracy of the obtained values was determined for the red clay samples with 30 wt% PbO, Bi_2_O_3_ and CdO instead of 10 wt%, to test whether increasing the amount of additives affected the reliability of the results. This figure has similar trends to the previous figure; namely, the difference between the XCOM and the experimental results was extremely small (within an acceptable experimental error). This once again proves that the experimental setup used in this work provides accurate data for red clay with both low and high amounts of PbO, Bi_2_O_3_, and CdO.

**Figure 5:**
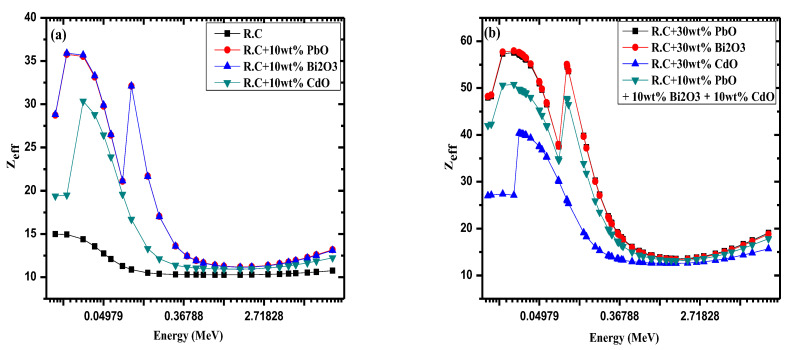
The effective atomic number as a function of energy, (**a**) *Z_eff_* of pure redl-clay as well as same the clay filled with 10%wt different oxides, and (**b**) *Z_eff_* of ball-clay filled with 30%wt different oxides.

The effective atomic number (*Z_eff_*) of pure red clay, and red clay with 10 wt% PbO, Bi_2_O_3_ and CdO is illustrated in Figure 5a, while Figure 5b shows the results for red clay with 30 wt% PbO, Bi_2_O_3_, and CdO, as well as for a red clay sample with 10 wt% of PbO, Bi_2_O_3_, and CdO (totaling 30 wt% metal oxide). Figure 5a demonstrates that pure red clay has the lowest *Z_eff_* out of the tested samples, which means that introducing any of these three oxides into the clay will greatly enhance its *Z_eff_*, and, consequently, its attenuation capability. In addition, the figure shows that the *Z_eff_* for red clay with 10 wt% CdO is lower than the *Z_eff_* of red clay with 10 wt% Bi_2_O and PbO, which is expected as Cd has a lower atomic number than Bi and Pb. Meanwhile, pure red clay has a *Z_eff_* value of about 10–15, 15 at the lowest tested energy and then smoothly decreasing down to a constant value. The first subfigure also revealed a peak for the CdO clay and two peaks for both the PbO and Bi_2_O_3_ clays. These peaks can be attributed to the k-absorption edges of Cd, Pb, and Bi. In Figure 5b, the *Z_eff_* for red clay with 30 wt% CdO is once again lower than the *Z_eff_* of the clays with 30 wt% PbO and Bi_2_O_3_, as well as the clay with 10 wt% CdO, 10 wt% PbO, and 10 wt% Bi_2_O_3_. One peak was observed for the red clay with 30 wt% CdO and two peaks for the other samples, which confirms the conclusion that these peaks occur because of the presence of Pb and Bi. In both figures it can be seen that the maximum *Z_eff_* occurs at the lowest tested energy, and that the minimum values occur in the moderate-energy range (which is due to the Compton scattering effect). When comparing the *Z_eff_* values for the clay sample with 10 wt% PbO to the sample with 30 wt% PbO, it can be seen that *Z_eff_* increases with an increase in PbO content, meaning that the *Z_eff_* of the red clay with 30 wt% PbO is greater than that of the sample with 10 wt% PbO. Therefore, adding more PbO to the clay samples improves the shielding ability of the red clay. This same conclusion applies to increased amounts of both CdO and Bi_2_O_3_.

**Figure 6:**
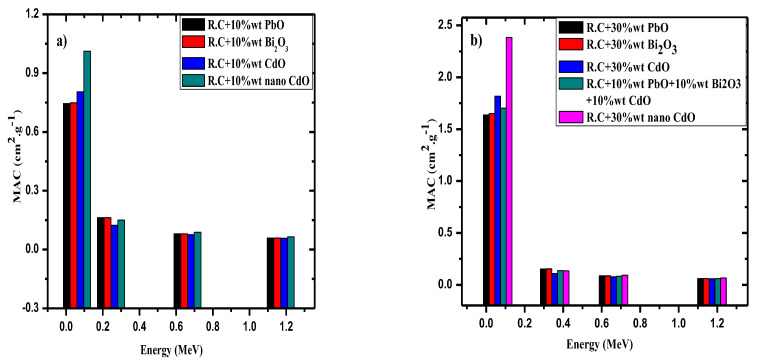
(**a,b**) The MAC for red clay filled with 10 wt% of different micro-oxides and 10 wt% CdO nanoparticles.

The MAC for red clay with 10 wt% PbO, Bi_2_O_3_, and CdO microparticles were compared with the MAC of red clay with 10 wt% CdO nanoparticles at four different energies (Figure 6a). This comparison tested the effect of decreasing particle size on the MAC of the red clays. The figure shows that the MAC of the red clay with 10 wt% nano-CdO was higher than the MAC of the clay containing microparticle samples. This difference is most evident at the lowest tested energy, and decreases as the energy increases. This result suggests that nanoparticles could be a useful way to enhance the shielding ability of current radiation-shielding materials.

Since the red clays containing nanoparticles outperformed the clays with microparticles, another red clay with 30 wt% nano-CdO was prepared, and the MAC for this sample was compared with that of the 30 wt% micro-PbO, Bi_2_O_3_, and CdO, as well as to that of a sample with 10 wt% micro-PbO, 10 wt% micro-Bi_2_O_3_, and 10% wt% micro-CdO. The results for these samples are graphed in Figure 6b. This figure shows that the MAC for the red clay with nano-CdO is higher than the MAC of the clay containing micro-PbO, CdO and Bi_2_O_3_, which is especially apparent at the first tested energy. Therefore, it can be concluded that one method to improve the shielding ability of materials is to introduce nanoparticles rather than using microparticles. Additionally, it can be said that nano-CdO can be used as an alternative to PbO to create a more environmentally friendly shielding material.

**Figure 7:**
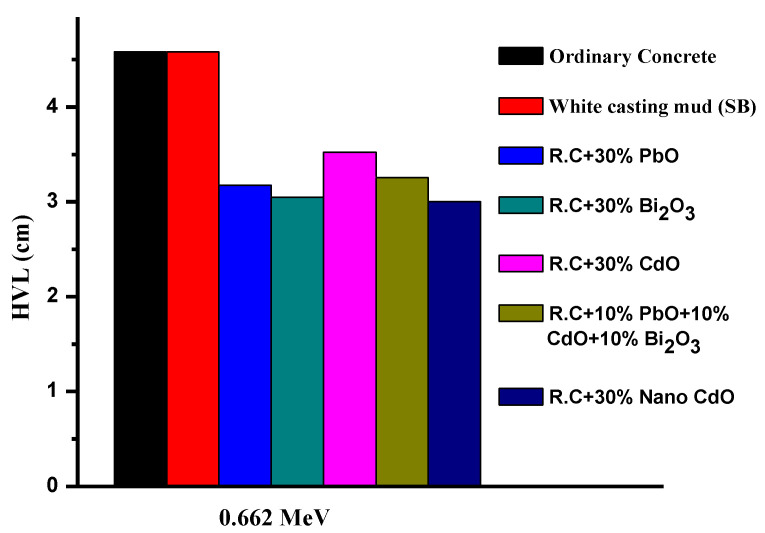
The HVL of the studied materials compared with ordinary concrete and white casting mud.

Finally, the present ceramic samples based on red clay were compared with two other materials used as a shielding material in nuclear facilities (concrete [40], and white ceramic [41]) as shown in Figure 7, where the HVL values were 4.581; 4.582; 3.175; 3.049; 3.525; 3.526; and 3.001 (cm) for concrete; white ceramic; R.C with 30 wt% PbO; R.C with 30 wt% Bi_2_O_3_; R.C with 30 wt% bulk CdO; R.C with 10 wt% PbO, 10 wt% Bi_2_O_3_ and 10 wt% CdO; and R.C with 30 wt% nano-CdO, respectively. The results indicated that the present ceramic-materials-based red clay has good radiation-shielding features.

## 4. Conclusions

In summary, this work started by collecting red clays from Aswan city in Egypt and blending them with various percentages of three oxides (PbO, Bi_2_O_3_ and CdO) with the aim of fabricating novel clay materials with enhanced gamma-radiation-shielding features. The MAC for the prepared materials was experimentally measured and compared with the theoretical results determined by XCOM. The measured and XCOM data agree to a very high degree of precision. Accordingly, the experimental setup used in this study can be reliably used to determine the shielding ability of the investigated clays. The *Z_eff_* of pure red clay, red clay with 10 wt%, and red clay with 30 wt% PbO, Bi_2_O_3,_ and CdO is reported. The *Z_ef_*_f_ results demonstrated that introducing any of these three oxides into clay will greatly enhance its *Z_eff_*, and, consequently, its attenuation capability. The *Z_eff_* for red clay with 30 wt% CdO is lower than the *Z_eff_* of the clays with 30 wt% PbO and Bi_2_O_3_, and the clay with 10 wt% CdO, 10 wt% PbO, and 10 wt% Bi_2_O_3_. Additionally, we found that the *Z_eff_* increases with an increase in PbO content, meaning that the *Z_eff_* of the red clay with 30 wt% PbO is greater than that of the sample with 10 wt% PbO. We compared the MAC of red clay with nano-CdO, to that of red clay with micro-PbO and micro-Bi_2_O_3_, to understand the influence of particle size on the attenuation ability of the red clays. We found that the MAC for the red clay with nano-CdO was higher than the MAC of red clay wih micro-PbO, CdO, and Bi_2_O_3_. Therefore, it can be concluded that one method to improve the shielding ability of materials is to introduce nanoparticles rather than using microparticles. Additionally, it can be said that nano-CdO can be used as an alternative to PbO to create a more environmentally friendly shielding material.

## Figures and Tables

**Table 1:** Chemical composition and densities of prepared ceramic-based red clay samples.

Sample	Weight Percentage (%)	Density (g·cm^−3^)	Weight Fraction of Elements (%)							
			Al	O	Si	Ti	Fe	Pb	Bi	Cd
Red clay (R.C)	100	1.982 ± 0.005	18,036	49,132	27,238	1.186	4.407	-	-	-
R.C + PbO	90:10	2.151 ± 0.003	16,232	44,932	24,512	1.067	3.964	9.283	-	-
R.C + PbO	70:30	2.602 ± 0.018	12,628	36,547	19,067	0.833	3.085	27.849	-	-
R.C + Bi_2_O_3_	90:10	2.150 ± 0.012	16,232	45,245	24.,512	1.068	3.964	-	8.970	-
R.C + Bi_2_O_3_	70:30	2.147 ± 0.005	12,627	37,487	19,068	0.833	3.086		26.910	
R.C + CdO	90:10	2.562 ± 0.008	16,232	45,460	24,512	1.066	3.964	-	-	8.754
R.C + CdO NPs	90:10	2.152 ± 0.017	16,320	45,372	24,475	1.054	3.976	-	-	8.791
R.C + CdO	70:30	2.565 ± 0.004	12,628	38,134	19,067	0.833	3.086	-	-	26,262
R.C + CdO NPs	70:30	2.599 ± 0.020	12,522	38,152	19,004	0.835	3.087	-	-	26,398
R.C + PbO + Bi_2_O_3_ + CdO	70:10:10:10	2.581 ± 0.011	12,628	37,389	19,067	0.833	3.086	9.283	8.969	8.754

**Table 2:** The activities and other specifications for point sources that are used in this study.

PTB Nuclide	Energy (keV)	Emission Probability	Initial Activity (kBq)	Reference Date	Uncertainty (kBq)
Am-241	59.52	35.9	259	1 January 2009	±2.6
Ba-133	80.99	34.1	275.3		±2.8
	356.21	21.4			
Cs-137	661.66	34.1	385		±4.0
Co-60	1173.23	99.9	212.1		±1.5
	1332.50	99.982			

## Data Availability

All data are available in the manuscript.mdpi.com/ethics. You might choose to exclude this statement if the study did not report any data.
